# Recent Advances in the Modulation of Pain by the Metabotropic Glutamate Receptors

**DOI:** 10.3390/cells11162608

**Published:** 2022-08-21

**Authors:** Mariacristina Mazzitelli, Peyton Presto, Nico Antenucci, Shakira Meltan, Volker Neugebauer

**Affiliations:** 1 Department of Pharmacology and Neuroscience, School of Medicine, Texas Tech University Health Sciences Center, Lubbock, TX 79430, USA; 2 Center of Excellence for Translational Neuroscience and Therapeutics, Texas Tech University Health Sciences Center, Lubbock, TX 79430, USA; 3 Garrison Institute on Aging, Texas Tech University Health Sciences Center, Lubbock, TX 79430, USA

**Keywords:** metabotropic glutamate receptor, mGluR, pain, pharmacology, neuroimmune signaling

## Abstract

Metabotropic glutamate receptors (mGluR or mGlu) are G-protein coupled receptors activated by the binding of glutamate, the main classical neurotransmitter of the nervous system. Eight different mGluR subtypes (mGluR1-8) have been cloned and are classified in three groups based on their molecular, pharmacological and signaling properties. mGluRs mediate several physiological functions such as neuronal excitability and synaptic plasticity, but they have also been implicated in numerous pathological conditions including pain. The availability of new and more selective allosteric modulators together with the canonical orthosteric ligands and transgenic technologies has led to significant advances in our knowledge about the role of the specific mGluR subtypes in the pathophysiological mechanisms of various diseases. Although development of successful compounds acting on mGluRs for clinical use has been scarce, the subtype-specific-pharmacological manipulation might be a compelling approach for the treatment of several disorders in humans, including pain; this review aims to summarize and update on preclinical evidence for the roles of different mGluRs in the pain system and discusses knowledge gaps regarding mGluR-related sex differences and neuroimmune signaling in pain.

## 1. Introduction

Persistent and chronic pain is a complex and debilitating disorder that affects millions of individuals and places a significant burden on the healthcare system [1,2]. Only limited therapeutic options are available, and they are frequently associated with severe side effects, creating a desperate need for new and effective analgesic strategies. 

As the most abundant excitatory neurotransmitter of the nervous system, glutamate regulates many physiological processes such as sensory processing, cognition, emotions, and learning and memory, but glutamatergic system dysfunctions have been linked to several diseases such as Parkinson’s and Alzheimer’s diseases, depression, anxiety, epilepsy, and pain [3,4]. Glutamate acts on ligand-gated ion channels (iGluR) that play a significant part in the glutamate-induced rapid response, as well as on G protein-coupled metabotropic glutamate receptors (mGluRs) that mediate the slower neuromodulatory response to glutamate. Thus far, eight mGluRs have been discovered and are classified into three groups based on their sequence similarity, signaling, and pharmacology [4,5,6,7,8]. The iGluR antagonists have been tested in several disease models, including pain; however, they have significant side effects such as memory loss, disorientation, and psychotic-like symptoms such as hallucinations and depersonalization [9], which has led to a focus on compounds acting on mGluRs as pharmacological tools to modulate glutamatergic transmission [7,10,11]. In addition to conventional mGluR ligands, a new line of compounds that can be activated or inactivated by light has been developed in the fairly new field of optopharmacology; they include caged compounds and photoswitchable ligands. Optopharmacology is being applied to the study of pain mechanisms and mGluR function [12,13]. 

**Figure 1:**
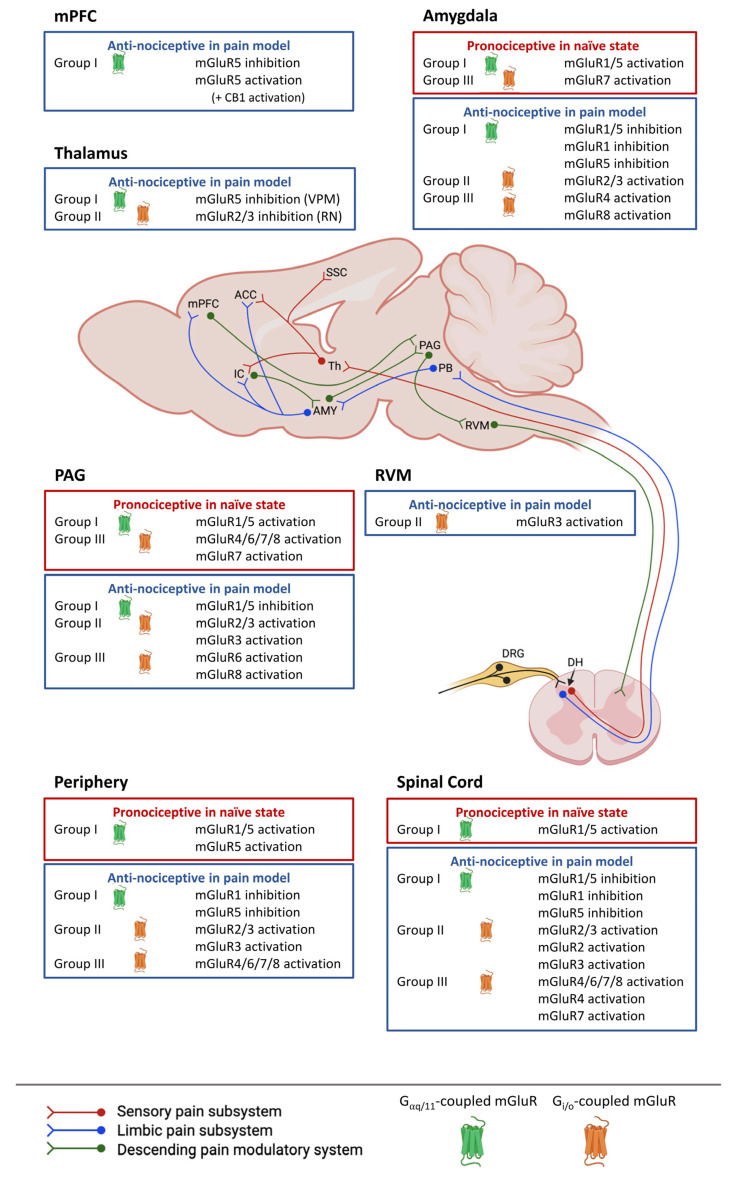
mGluR signaling within the pain system. Nociceptive information is carried from the periphery into the CNS via first-order neurons in the dorsal root ganglia (DRG). Axons from second-order neurons in the spinal dorsal horn (DH) carry nociceptive information to different brain regions involved in sensory and affective aspects of pain; these pain pathways include the spinothalamic tract (STT, shown in red) that connects to the thalamocortical system of posterior thalamic (Th) nuclei and somatosensory cortex (SSC) for sensory-discriminative aspects of pain. Note that STT also connects to medial thalamic (Th) nuclei and so-called paralimbic areas such as the insular cortex (IC) and medial prefrontal cortex (mPFC) with anterior cingulate cortex (ACC) for complex cognitive-evaluative processes. The SPbA tract relays nociceptive information via the parabrachial (PB) nucleus in the brainstem to limbic regions such as the amygdala (AMY). The AMY integrates nociceptive and multimodal information to create emotional-affective states through connections with IC, mPFC and ACC. Brain regions that engage top-down descending pain modulation (shown in green) include efferents from the mPFC, ACC and AMY that synapse onto periaqueductal gray (PAG) neurons, which then project to the rostral ventromedial medulla (RVM) to modulate spinal nociceptive processing via descending monoaminergic projections. The reported effects of mGluR signaling within the pain system are shown by the red (pronociceptive; facilitatory effect on pain-like behaviors in a naïve state) and blue (anti-nociceptive; inhibitory effect on pain-like behaviors in pain models) boxes for each region. G_αq/11_-coupled receptors are indicated in green and G_i/o_-coupled receptors in orange. ACC, anterior cingulate cortex; AMY, amygdala; CB1, cannabinoid receptor type 1; DH, dorsal horn of spinal cord; DRG, dorsal root ganglion; IC, insular cortex; mPFC, medial prefrontal cortex; PAG, periaqueductal gray; PB, parabrachial nucleus; RN, reticular nucleus of the thalamus; RVM, rostral ventromedial medulla; SSC, somatosensory cortex; Th, thalamus; VPM, ventral posteromedial nucleus of the thalamus. Created with BioRender.com.

Here, we briefly summarize and update the key effects of mGluR compounds tested in clinical trials and review evidence from preclinical studies about the role of mGluRs in pain modulation (Figure 1). We also address knowledge gaps about the potential mGluR-related sex differences and neuroimmune signaling in the context of pain.

## 2. mGluRs and Clinical Trials in The Context of Pain

mGluRs have been explored as clinical therapeutic targets for various neurological disorders. For example, GET 73 (N-[(4-trifluoromethyl)benzyl]4-methoxybutyramide, structurally related to gamma-hydroxybutyric acid) is an mGluR5 negative allosteric modulator (NAM) that has been proposed as a treatment strategy for alcohol use disorder (AUD) [14,15] and obesity [16]. Both single and repeated oral doses of GET 73 were shown to be safe and without significant side effects in healthy volunteers in a Phase I clinical trial [17], supporting its further development as a treatment for these disorders. GET 73 advanced to a Phase II clinical trial on central glutamate and GABA levels and brain activity in AUD patients (NCT03418623). Other mGluRs have been the targets of antipsychotic drug development. Group II mGluR agonists have been tested for the treatment of schizophrenia [18], though some clinical trials have reported ineffectiveness and undesirable side effects. A group II mGluR agonist prodrug (LY2140023, pomaglumetad methionil) did not show any greater reduction in the negative symptoms of schizophrenia compared to placebo [19] or standard of care [20] treatments in Phase II and III trials, but there was some evidence for an antipsychotic effect in a specific genetically-defined subpopulation. Therefore, there is continued discussion about these drugs as potential clinical alternatives following further investigation into their cellular and molecular actions on different subpopulations of schizophrenia patients across different disease stages [21,22]. mGluR modulators have also been explored for the treatment of mood disorders, though clinical trials for both an mGluR5 NAM (basimglurant) and an mGluR2/3 NAM (RG1578; decoglurant) found little to no antidepressant effects compared to placebo [23,24]; these examples serve as important illustrations of the clinical viability of mGluR drug discovery and development in various disorders that may also be relevant for pain conditions.

Much less is known about the role of mGluRs in pain management in the clinical setting. Although anti-nociceptive effects of these drugs have been demonstrated in multiple animal pain models as discussed in this review, no pharmacological tools that target mGluRs have been successfully developed as analgesics in patients. One potential candidate that briefly emerged as a therapeutic option was the investigational drug fenobam, a selective and non-competitive mGluR5 antagonist that was originally developed as a non-benzodiazepine anxiolytic [25]. Whereas robust antinociception with fenobam treatment has been demonstrated in preclinical mouse models of inflammatory [26,27], neuropathic [28,29], and visceral [30] pain, minimal analgesic effects of fenobam were found in a human experimental pain model of cutaneous sensitization [31]. Despite this inconsistency, more investigation into the effectiveness and profile of glutamatergic drugs in clinical pain conditions is warranted. One important consideration when evaluating the clinical translatability of such drugs is the blood–brain barrier (BBB) permeability. Though restrictive BBB transport mechanisms provide neural tissue with a protective barrier from pathogens and toxins under physiological conditions, they also pose a limitation to drug design in central nervous system (CNS) disorders [32]; however, BBB dysfunction has been reported in pain and neuroinflammatory conditions [33,34,35,36], where the resultant BBB leakage and increased permeability may favor increased drug access to the CNS. AsTablepreclinical pain studies continue to explore the biological actions of mGluRs and subtype selective agents, further insight will be gained regarding underlying mechanisms, site(s) of action, and particular conditions that may influence the effectiveness of mGluR drugs in human pain patients.

## 3. Group I mGluRs in Preclinical Pain Models 

Group I mGluRs (GluR1 and mGluR5) are predominantly expressed postsynaptically and positively regulate phospholipase C (PLC) by coupling to G_αq/11_ proteins. Therefore, blockade of mGluR1/5 has been used as the preferential intervention to mitigate pain. Systemic intraperitoneal (i.p.) application of selective mGluR1 NAMs (EMQMCM) was ineffective in changing the thermal thresholds in naïve animals [37], but had antinociceptive properties in both phases of the formalin test (EMQMCM, FTIDC) [37,38], in the skin incision model of post-operative pain (A-841720, A-794282, A-794278, and A-850002) [39], and in inflammatory (intraplantar injection of complete Freund’s adjuvant (CFA) or intra-knee joint injection of monoiodoacetate) and neuropathic (LY456236 or A-841720) (spinal nerve ligation, SNL, or chronic constriction injury, CCI) [40,41] pain models. Similarly, systemic (i.p. or oral) injection of mGluR5 NAMs (MPEP, MTEP or fenobam) had no effects on thermal sensitivity under normal condition [37,42] but decreased nociceptive responses in the formalin test [26,28,37,38,40,43], in the skin incision pain model [43], and in inflammatory (CFA, carrageenan, visceral [induced by acetic acid i.p. injection]) [26,27,42,43] and neuropathic (CCI, SNL, and chemotherapy [induced by intravenous, i.v., injection of vincristine)] [40,42,43,44,45] pain models. MPEP and MTEP (i.v.) reduced the visceromotor responses evoked by colorectal distension in awake normal rats [46]. Based on preclinical studies (see Table 1), systemically applied mGluR1 and/or mGluR5 antagonists or NAMs are effective in rodent pain models representing an attractive strategy to restore physiological functions in pain condition. 

### 3.1. Preclinical Behavioral Studies

Periphery. Intraplantar (i.pl.) injection of a group I mGluR agonist (DHPG) or an mGluR5 agonist (CHPG) induced mechanical and thermal hyperalgesia in normal animals [47,48,49], suggesting that activation of GluR1/5 has pronociceptive effects that mimic a pain condition. Microinjection of mGluR5 NAM (MPEP), but not mGluR1 NAM (CPG), resulted in a dose-dependent inhibition of the agonist (exogenous glutamate, DHPG or CHPG)-induced mechanical hyperalgesia, providing evidence for a mGluR5-, rather than mGluR1-, mediated hyperalgesic action in peripheral tissues [47]; these results were consistent with a different study analyzing mGluR5 contribution in craniofacial nociception, where mGluR5 NAM (MPEP), but not mGluR1 NAM (CPCCOEt), administered into the masseter muscle reverted DHPG-induced mechanical hypersensitivity in normal rats, which was reduced by a specific TRPV1 antagonist (AMG9810) [50]. Injection of an mGluR1 NAM (CPCCOEt or LY367385) or mGluR5 NAM (MPEP) into the hindpaw had no effects in non-sensitized animals but abolished DHPG- or capsaicin-induced thermal hyperalgesia [48,49] and decreased the nociceptive behaviors in the late phase of the formalin test [49], while a different mGluR5 NAM (Raseglurant) reduced the pain responses 5 min after formalin (i.pl.) injection [78]. Peripheral (i.pl.) administration (pretreatment) of mGluR1 (CPCCOEt or LY36738) or mGluR5 (MPEP or SIB1893) blockers improved the mechanical allodynia, measured by the application of increasing air-puffs pressure and generated by the injection of interleukin-1β (IL-1β, s.c.) into a rat vibrissa pad [51]. Similar effects were observed when MPEP was peripherally (i.pl.) administered in CFA and carrageenan inflammatory models [47] as well as in the skin incision model of pain [52]. Finally, peripheral blockade of mGluR5 achieved by the photoreaction of JF-NP-26 (systemic), an inactive photoswitchable mGluR5 NAM, with violet light (385 nm) applied on the hindpaw to generate the activate molecule diminished nociceptive behaviors in both phases of the formalin test [53]; these findings (see Table 1) suggest that mGluR5 rather than mGluR1 drives pain-related responses in peripheral tissues and that mGluR5 blockade has consistent antinociceptive properties when mGluR5 NAMs are applied locally in several models of pain.

Spinal cord. Intrathecal administration of a group I mGluR agonist (DHPG or 1S,3R-ACPD) induced nociceptive behaviors [54,55,59,60,61], cold hypersensitivity [56], mechanical allodynia and thermal hyperalgesia [57,58,62] in normal animals; these effects were blocked by pretreatment with mGluR1 NAM (CPCCOEt) [59] or mGluR5 NAM (MPEP) [55,56,59] and correlated with activation of extracellular signal-regulated kinases ERK1 and ERK2 in the spinal cord [59], suggesting that activation of spinal group I mGluRs mediates nociceptive responses. Interestingly, the effects of DHPG were lost in K^+^ channel subunit Kv4.2 knock-out mice, providing evidence that Kv4.2 expression is necessary for DHPG-induced nociceptive behaviors [61]; moreover, DHPG (i.th.) potentiated, in a dose-dependent fashion, the nociceptive responses in the formalin test [63] and in the CFA inflammatory pain model [60]; these facilitatory effects were reverted by a group I mGluR antagonist (S-4C3HPG) [63]. Surprisingly in a recent study the intrathecal application of a group I mGluR agonist (1S,3R-ACPD) had antinociceptive properties in the CFA model of inflammatory pain, suggesting a potential switch of the receptor functions in pathological conditions [58]. Spinal blockade of group I mGluRs by the application of an antagonist (LY393053, i.th.) had weaker effects than an mGluR5 NAM (fenobam, i.th.) on glutamate-induced behaviors in neuropathic (spared nerve injury, SNI) rats [64], while both had antinociceptive properties in an inflammatory (CFA) pain model [65]. In a different model of neuropathic pain (CCI), intrathecal application of group I antagonists (S-4C3HPG or AIDA) decreased mechanical allodynia and cold hyperalgesia [66,67], whereas in the CFA model, AIDA (i.th.) ameliorated mechanical withdrawal thresholds, but failed to change thermal responses [68]. Selective mGluR1 NAM (CPCCOEt, i.th.) and mGluR5 NAM (MPEP, i.th.) reduced pain behaviors in both phases of the formalin test [59] and mechanical allodynia, but not cold hypersensitivity, in a neuropathic (CCI) condition [56,67] and ameliorated mechanical hyperalgesia in paclitaxel- (chemotherapy) [69] and streptozotocin- (diabetic) [70] induced neuropathic rats. Thus, at the spinal cord level, activation of mGluR1 and/or GluR5 is pronociceptive in the absence of pain, suggesting that these receptors may contribute to the development of the pain pathology, whereas blockade of mGluR1/5 (or each subtype) has beneficial effects in the pain state; it should be noted that one study [60] reported antinociceptive properties of a group I mGluR agonist applied spinally in an inflammatory pain model (see Table 1). 

Brain. Administration of a group I mGluR agonist (DHPG), into the central nucleus of amygdala (CeA) promoted mechanical hypersensitivity [71,72], and increased the duration of the vocalizations evoked by noxious mechanical stimulation of peripheral (knee joint) or visceral (colorectal distension) tissues [72] in normal naïve animals; these effects were blocked by the intra-amygdala co-administration of an mGluR5 NAM (MTEP), but not an mGluR1 NAM (LY367385), and involved inositol-1,4,5-trisphosphate (IP3) signaling and reactive oxygen species formation [72]. Application of an mGluR1 NAM (CPCCOEt) into the CeA by microdialysis inhibited the enhanced emotional-affective responses (vocalizations in the ultrasonic and audible ranges) recorded during (vocalizations organized at the medullary level) and immediately after (vocalization afterdischarges generated in the limbic forebrain) innocuous and noxious compressions of the arthritic knee joint in the kaolin/carrageenan (K/C) model, whereas an mGluR5 NAM (MPEP) decreased only the vocalization afterdischarges [75]. Therefore, mGluR1 and mGluR5 in the amygdala have different contributions to supraspinally organized pain behaviors. MPEP stereotaxically administered into the dorsal striatum as an offsite control did not change the stimulus-evoked vocalizations in arthritic animals [75]. CPCCOEt, but not MPEP, injected into the basolateral amygdala (BLA) resulted in antihyperalgesic effects on mechanosensitivity in the intraplantar carrageenan-induced inflammatory pain model [76]. Viral vector-mediated (lentivirus EF1α-mGluR5-IRES-Zsgreen1) bilateral overexpression of mGluR5 in the pre-limbic (PL) division of the medial prefrontal cortex (mPFC) decreased the mechanical withdrawal thresholds in normal rats [77], mimicking a pain state. Bilateral injection of mGluR5 NAMs (MPEP or MTEP) into the PL cortex resulted in antinociceptive effects in the SNL model of neuropathic pain [77]. Interestingly, a photoswitchable active mGluR5 NAM (Alloswitch-1) injected into the amygdala ameliorated the mechanical allodynia in CFA injected mice, and these effects were abolished by the subsequent local (intra-amygdala) inactivation of the mGluR5 compound through focal illumination with violet light (385 nm) [78], providing evidence for the effectiveness of optopharmacology as a potential therapeutical strategy for pain relief. Systemic (i.p.) application of a normally inactive photoswitchable mGluR5 NAM (JF-NP-26) also reduced nociceptive behaviors in both phases of the formalin test and increased the mechanical thresholds in a neuropathic (CCI) pain condition when violet light (385 nm) was delivered into the ventral posteromedial nucleus (VPM) of the thalamus for local photoactivation of the compound [53]. The application of a group I agonist (DHPG) into the dorsolateral periaqueductal gray (dlPAG) facilitated nociceptive behavior in the hot plate test under normal conditions, and this effect was blocked by a group I antagonist (AIDA), which had inhibitory properties when injected alone [73]. Intra-dlPAG DHPG decreased the nociceptive responses in the late phase of the formalin test [74]. The concurrent activation of mGluR5 and cannabinoid (CB1) receptors in the infralimbic (IL) division of the mPFC, but not in the anterior cingulate cortex (ACC), by the co-administration of a positive allosteric modulator (PAM) selective for mGluR5 (VU0360172) and a CB1 agonist (ACEA) had inhibitory effects on the mechanosensitivity and vocalizations evoked by noxious compression of the injured knee joint in the K/C monoarthritis pain model [79]. According to these results (see Table 1), mGluR1 and mGluR5 have different functions depending on the brain area targeted. 

Taken together, evidence from behavioral studies suggests that mGluR5 has a critical contribution to nociceptive functions along the pain neuroaxis. The subtype-specific role in brain areas involved in descending pain modulation (PAG, RVM) and at the cortical level remains to be determined. 

### 3.2. Preclinical Electrophysiological Studies

Periphery. To the best of our knowledge, only a few studies have addressed the role of group I mGluRs in the periphery in pain processing. For instance, a group I mGluR agonist (DHPG) generated an increase in the inward current responses of dorsal root ganglion (DRG) cells in whole ganglia preparations obtained from naïve rats [80,81] and enhanced Ca^2+^ signals in DRG mixed culture from normal rats [80]. The facilitatory effects were blocked by a non-specific group I antagonist (DL-AP3) [80,81] and were greater in small diameter DRG neurons from CCI than from normal animals [81]; moreover, DHPG incubation (2 h) reduced both AMPA- and NMDA-induced inward currents in naïve and pain (CCI) conditions while causing an increase of kainic acid-induced inward currents only in naïve DRGs [81]. An mGluR5 NAM (MTEP) reduced the responses of pelvic afferents to colonic distension and of splanchnic afferents to colonic probing (von Frey) in an in vitro mouse colonic afferent preparation [46]. Based on this relatively thin evidence, mGluR5 seems to contribute to visceral pain responses, while both mGluR1 and mGluR5 can modulate peripheral neuronal changes related to nociceptive processing. More information is needed before peripheral mGluR1 and/or mGluR 5 can be considered as a target for pain mitigation. 

Spinal cord. In vivo electrophysiological studies in the spinal dorsal horn of anesthetized adult rats showed that spinal application of a group I mGluR agonist (1S,3R-ACPD) increased C-fiber-evoked field potentials under normal conditions through an L-type Ca^2+^ channel-mediated mechanism [58]. In contrast, 1S,3R-ACPD generated inhibitory responses in CFA-injected anesthetized animals that were independent of L-type Ca^2+^ channels but involved GABA-A and glycine inhibitory transmission [58]. Activation of mGluR5 (CHPG) mimicked the inhibitory effects of the group I agonist, while mGluR5 blockade (MPEP) had no effects in the inflammatory pain (CFA) model, but reversed the 1S,3R-ACPD-induced inhibitory effects, suggesting that mGluR5 mediates beneficial inhibitory effects of a group I mGluR agonist in the spinal cord in pain conditions [58]. Single-unit recordings in the spinal dorsal horn of anesthetized rats found that a group I agonist (DHPG, i.th.) had mixed effects on the neuronal activity of wide dynamic range (WDR) neurons, which respond more strongly to noxious than innocuous peripheral mechanical stimulation, under normal and inflammatory pain (carrageenan, s.c.) conditions [82]. Likewise, intrathecal DHPG had concentration-dependent excitatory and inhibitory effects on evoked activity of dorsal horn neurons in anesthetized monkeys (Macaca fascicularis) under normal conditions, whereas an mGluR5 agonist (CHPG) had inhibitory effects [83]. In contrast, peripheral microinjection of CHPG induced spontaneous firing of dorsal horn WDR neurons in anesthetized rats that was inhibited by the co-application of an mGluR5 NAM (MPEP), which had no effects when administered alone [47]. Intrathecal application of an mGluR1 antagonist (AIDA) or NAM (CPCCOEt) reversed capsaicin-induced increase in activity of dorsal horn WDR neurons in anesthetized monkeys (Macaca fascicularis) [83]. Intravenous administration of an mGluR1 NAM (A-841720) decreased the responses of WDR neurons to mechanical stimulation in a rat neuropathic pain model (SNL) [41]. Ex vivo recordings of spinal cord dorsal horn neurons (approximately lamina III) in primary cultures from normal animals showed that bath application of a group I agonist (DHPG) increased neuronal excitability and decreased the peak amplitude of A-type currents through ERK activation [61]. The DHPG-induced effects were lost in Kv4.2 knock-out mice and were blocked by the co-application of an mGluR5 NAM (MPEP), but not mGluR1 NAM (LY367385) into the bath, suggesting that Kv4.2 expression and mGluR5 are necessary for the DHPG-mediated neuronal modulation [61]; moreover, application of DHPG failed to change Ca^2+^ release in spinal cord dorsal neurons whereas a broad-spectrum group I agonist (quisqualate) increased nuclear Ca^2+^ increase, which was prevented by an mGluR5 NAM (Fenobam) [64]. Recordings of spinal lamina II neurons in slice preparations obtained from normal rats showed that DHPG applied into the bath increased the frequency of miniature excitatory postsynaptic currents (mEPSCs in TTX) and the amplitude of EPSCs evoked by dorsal root stimulation and reduced the paired pulse ratio (PPR) of evoked EPSCs, mimicking the neuronal changes observed in the paclitaxel-induced neuropathic pain model [69]; these effects were reversed by the administration of an mGluR5 (MPEP), but not mGluR1 (LY367385), blocker. The results of this study suggest that the pharmacological effects of the group I mGluR agonist were mediated by presynaptic mGluR5 rather than mGluR1 [69]; moreover, MPEP normalized the paclitaxel-induced changes of miniature and evoked EPSCs in spinal neurons, but had no effects in vehicle control animals, pointing to a tonic mGluR5 activation in the pain state, which was associated with PKC and NMDA signaling [69]. Similar observations were made in spinal cord slice preparations from diabetic (streptozotocin) neuropathic rats where MPEP reduced the enhanced EPSC peak amplitude evoked from primary afferents and spontaneous (sEPSC) frequency of dorsal horn neurons, whereas CPCCOEt was ineffective [70]. Although, some of the results reported in these electrophysiological studies are mixed, if not contradictory, evidence generally points to a critical role of mGluR5 in spinal nociceptive processing. 

Brain. Hyperactivity in the amygdala leads to deactivation of mPFC (IL and PL), and mitigating this inhibition and restoring mPFC output inhibits amygdala activity and pain-related behaviors (see *Preclinical behavioral studies*) [84,85]. In contrast, the ACC has largely pronociceptive function [86]. In vivo electrophysiological single-unit recordings of CeA neurons showed that the activation of group I mGluRs (DHPG) and mGluR5 (CHPG) in the CeA enhanced the neuronal responses evoked by mechanical stimulation of peripheral tissues in normal rats, while only DHPG had facilitatory effects in arthritic (K/C pain model) rats [87]. An mGluR1 NAM (CPCCOEt) administered stereotaxically into the CeA reduced the evoked responses of CeA neurons in the arthritis pain state, while an mGluR5 NAM (MPEP) had inhibitory properties in both normal and pain conditions, providing evidence for a change of mGluR1 function in pain [87]. Ex vivo patch-clamp recordings in brain slices from normal rats showed that a group I mGluR agonist (DHPG) increased cellular excitability of CeA neurons in the latero-capsular division (CeLC), which is defined as the “nociceptive amygdala” [72,85] and potentiated the amplitude of monosynaptic EPSCs evoked by electrical stimulation of BLA or parabrachial nucleus (PB) that provides nociceptive input to the CeA [88]; these effects were mimicked by mGluR5 activation (CHPG) [72,88] and were prevented by the bath application of an mGluR5 NAM (MTEP) but not by an mGluR1 antagonist (LY367385); MTEP and LY367385 had no effects when applied alone [72]. The DHPG-induced facilitation was mediated by IP3-dependent ROS formation and downstream ERK and protein kinase A (PKA), but not PKC, signaling [72]. mGluR1 blockade by bath application of an antagonist (LY367385) or NAM (CPCCOEt) decreased the enhanced EPSCs and restored the reduced glutamatergic-driven synaptic inhibition (IPSCs) at the BLA- and PB-CeLC synapses in arthritic (K/C model) rats, whereas no effects were observed under normal conditions [72,88]. Conversely, mGluR5 NAMs (MTEP or MPEP) decreased excitatory transmission and increased inhibitory transmission at the BLA-CeLC synapses in both conditions, suggesting a critical contribution of mGluR1 to pain-related neuronal changes in the amygdala network [89]; moreover, the analysis of sEPSCs/mEPSCs and sIPSCs/mIPSCs revealed that mGluR1 acted presynaptically and mGluR5 postsynaptically at BLA-CeLC synapse [89]. 

In vivo electrophysiology single-unit recordings of individual neurons in the IL and PL divisions of the mPFC in anesthetized rats showed that administration of a group I mGluR agonist (DHPG) into the BLA resulted in a decrease of evoked activity of mPFC neurons that responded with an excitatory response to noxious mechanical stimuli [76]. Decreased responsiveness of mPFC neurons was also observed in electrophysiology in vivo studies in rat models of arthritis pain (K/C model) [90,91,92] and inflammatory pain (intraplantar carrageenan or formalin) [76]. The mPFC deactivation was reversed by the intra-BLA application of an mGluR1 NAM (CPCCOEt), but not an mGluR5 NAM (MPEP) [76], suggesting that mGluR1 in the amygdala play a critical role in driving mPFC deactivation. mGluR1 in the mPFC is also involved in pain-related mPFC deactivation, because single-unit recordings of PL pyramidal cells in anesthetized rats found that an mGluR1 antagonist (LY367385), but not MPEP, reversed the decrease of background and evoked activity in the K/C arthritis pain model. Blockade of mGluR1 or mGluR5 in the PL under normal conditions had no effect, but DHPG administered into the PL in normal animals decreased background and evoked activity of PL neurons through a GABA-A receptor dependent mechanism. Interestingly, activation of mGluR5 in the IL with a PAM (VU0360172) increased background and evoked activity of IL neurons under normal conditions, whereas in the K/C arthritis pain model co-activation of endocannabinoid CB1 receptor with ACEA was required to restore the facilitatory effects of VU0360172 [92]. Importantly, co-activation of mGluR5 and CB1 in the IL inhibited the pain-related increase of background and evoked activity of amygdala CeLC neurons [92]. Underlying synaptic mechanisms were identified in an electrophysiological ex vivo study in mPFC brain slices [79]. VU0360172 increased synaptically evoked spiking evoked by the electrical and optical stimulation of BLA input in IL pyramidal cells in brain slices from normal rats [79]. The facilitatory effect was abolished by the intracellular administration of an inhibitor of the major 2-arachidonoylglycerol synthesizing enzyme diacylglycerol lipase (tetrahydrolipstatin; THL) or a CB1 receptor antagonist (AM251), suggesting that cannabinoid-mediated mechanisms are required for the mGluR5-induced effects [79]. As in the in vivo studies, VU0360172 had no effects in IL neurons in slices from arthritic rats (K/C model), but co-application of ACEA or inhibitors of postsynaptic 2-AG hydrolyzing enzyme ABHD6 (intracellular WWL70) or monoacylglycerol lipase MGL (JZL184), or by blocking GABAergic inhibition with intracellular picrotoxin restored the effects of VU0360172 and as well as CB1-dependent depolarization-induced suppression of synaptic inhibition (DSI) to increase infralimbic output [79]. Stereotaxic coadministration of VU0360172 and ACEA into the ACC had no effects [79]. The data suggest that the inverse relationship between amygdala (BLA) and mPFC that permits uncontrolled amygdala hyperactivity in pain is mediated by mGluR1 activation in BLA and mPFC and by dysfunction of mGluR5-CB1 signaling in mPFC. 

Interestingly, mGluR5 blockade (MPEP) in the lateral entorhinal cortex (LEC) reduced the amplitude and slope of field potentials in the hippocampal dentate gyrus (DG) in anesthetized normal and neuropathic (SNI) mice, pointing to a tonic role of mGluR5 in hippocampal processing [93]; however, MPEP had no effect on field potentials in mice treated systemically for 15 days with palmitoylethanolamide (PEA), an endocannabinoid anandamide congener associated with increased 2-AG levels. MPEP and PEA reversed the impaired LTP at the LEC-DG pathway after theta-burst stimulation in SNI mice without affecting LTP in naïve mice, but the effects of MPEP on LTP were occluded in PEA-treated SNI, suggesting that blocking mGluR5 is a suitable approach to rescue hippocampal dependent abilities and that mGluR5 perhaps shares actions with the endocannabinoid system.

Based on the body of work reported here (for behavioral data see Table 2), changes in mGluR1 functions in pain conditions suggest an important contribution whereas the role of mGluR5 appears to be more complex and region specific and may involve interactions with the endocannabinoid system.

## 4. Group II mGluRs in Preclinical Pain Models

Group II mGluRs, which include mGluR2 and mGluR3, are G_i/o_ coupled receptors and promote the inhibition of adenylyl cyclase upon activation, decreasing the production of cAMP [120]; they are found throughout the nervous system, including regions and circuits involved in nociceptive signaling, pain modulation and emotional responses [121,122]. In preclinical studies, the activation of group II mGluRs by the systemic administration of selective agonists has consistently shown antinociceptive properties in several models of pain [4,5,6,123,124]. Systemic application (i.p.) of group II mGluR agonists (LY354740, LY379268, or LY389795) ameliorated nociceptive behaviors in the formalin test [98], decreased thermal hyperalgesia in rats after the injection of carrageenan into the paw or tail [96], and increased the mechanical withdrawal thresholds in monoarthritis (kaolin/carrageenan) [97] and neuropathic (SNL, CCI) [45,98] pain models. Similarly, analgesic effects were observed when a prodrug (LY2969822) for the group II mGluR agonist LY2934747 was orally administered in inflammatory (formalin, capsaicin, CFA), postsurgical (plantar incision), visceral (colorectal distension), and neuropathic (SNL) preclinical pain models [99]. An important knowledge gab exists about the role of group II mGluRs in pain-related neuroimmune signaling, although the involvement of these receptors with non-neuronal elements has consistently been reported in other conditions (see 6.).

A considerable number of studies have investigated the subtype-specific contributions of mGluR2 and mGluR3 in different pain conditions, but a clear picture has yet to emerge. Studies on subtype-specific actions in different regions of the pain system will be discussed below. Systemic (i.p.) administration of LY379268 resulted in antinociceptive effects in the formalin pain model in mGluR3, but not mGluR2, knock-out mice [100], pointing to an mGluR2 subtype-specific contribution. Endogenous activation of group II mGluRs with systemic (i.p.) application of N-acetylcysteine (NAC), activator of the L-cysteine/L-glutamate membrane exchanger (Sxc-) expressed on the astrocyte membrane [125,126], reduced the nocifensive reflex in the tail flick test in normal mice [94]; moreover, systemically (i.p.) administered NAC ameliorated nociceptive behaviors in the late phase of the formalin test, and mechanical hypersensitivity (von Frey) in inflammatory (CFA) and neuropathic (CCI) pain models, through a mechanism that involved increased endogenous activation of mGluR2 because NAC worked in mGluR3- but not mGluR2- knockout mice [95]. Repeated systemic (s.c.) injections of L-acetylcarnitine (LAC), an epigenetic drug that potentiates the transcription the gene encoding for mGluR2 (GRM2), reduced thermal hypersensitivity in sham control rats and mechanical allodynia (von Frey) in neuropathic (CCI and diabetes induced by streptozotocin) and inflammatory (CFA) pain conditions through a mechanism that involved increased expression of mGluR2 in the dorsal horn and DRG in both conditions [101,103]. Oral application of LAC together with ultra-micronized formulation of palmitoylethanolamide (PEA), a well-known analgesic fatty acid, significantly improved thermal hyperalgesia after carrageenan injection (i.pl.), while each drug alone failed to mitigate the pain responses, providing evidence for a synergistic mechanism [102]. Through a similar epigenetic mechanism as LAC, systemic (s.c.) application of histone deacetylase (HDAC) inhibitors (suberoylanilide hydroxamic acid, SAHA) improved the nociceptive behaviors in the second phase of the formalin test [104]. To better understand mGluR3 functions, the neuropeptide N-acetylaspartylglutamate (NAAG), a preferential activator of mGluR3 [127,128], or NAAG peptidase inhibitors that block NAAG degradation [129] have been employed. Systemic (i.v. or i.p.) administration of NAAG peptidase inhibitors (ZJ-11, ZJ-43 or 2-PMPA) had antinociceptive effects in both phases of the formalin test and anti-allodynic (von Frey) properties in the partial sciatic nerve ligation (PSNL) model of neuropathic pain [105,106]. 

Systemic activation of group II mGluRs has consistently produced beneficial effects in several pain models (see Table 2). Evidence from studies addressing subtype-specific roles in pain modulation points to a critical inhibitory function of mGluR2, which could also be due to the greater number of studies addressing the mGluR2 rather than mGluR3 subtype (see Table 2).

### 4.1. Preclinical Behavioral Studies

Periphery. Subcutaneous hindpaw intraplantar (i.pl.) injection of group II mGluR agonists (LY314582, 2R,4R-APDC or L-CCG-I) had no effects on thermal- and mechanical thresholds under normal conditions, suggesting that mGluR2/3 were not involved in peripheral nociception [47,48,107,109,110]. Administration of group II mGluR agonists (2R,4R-APDC or SLx-3095-1, the racemate of the highly selective group II mGluR agonist LY379268) into the hindpaw also had no effects on the mechanical and thermal sensitivity in normal rats and mice, but ameliorated prostaglandin E_2_ (PGE_2_)-induced thermal hyperalgesia, PGE_2_-, carrageenan- and inflammatory soup (IS)- induced mechanical allodynia, as well as nociceptive responses in both phases of the formalin test and in the capsaicin pain model [107,108,109,110,111,112]; moreover, pre-treatment (s.c.) with group II agonists (2R,4R-APDC and DCG4) improved the mechanical allodynia (to air-puffs pressure) induced by IL-1β subcutaneously injected into the rat vibrissa pad [51] and reduced the weight load deficits and the mechanical hypersensitivity (von Frey) in a carrageenan-induced arthritic pain model [113]. The inhibitory effects of the mGluR2/3 agonists were reversed by the co-application of a potent group II antagonist (LY341495) [51,107,109,110,111]. Importantly, the intraplantar injection of group II antagonists alone (LY341495 or APICA) prolonged the PGE_2_- and carrageenan-induced mechanical allodynia (von Frey) and exacerbated the nociceptive behaviors in the capsaicin model, providing evidence for the endogenous activation of mGluR2/3 in inflammatory pain conditions [110,112]. Although antinociceptive properties of peripheral group II mGluRs activation have been reported consistently in rodents, there may be differences in humans [130] (see “*Preclinical electrophysiological studies*”). With regard to subtype-specific actions, peripherally (s.c.) administered NAAG or NAAG peptidase inhibitors (ZJ-43 or 2-PMPA) decreased mechanical allodynia (von Frey) in the carrageenan inflammatory model and pain responses in both phases of the formalin test [108], pointing to a modulatory function of mGluR3. The results suggest that group II mGluR activation in peripheral tissues has beneficial inhibitory properties in several pain models, but not under normal conditions (see Table 2).

Spinal cord. Mixed effects of group II mGluR activation were reported under normal conditions. Spinal (i.th.) administration of group II agonists (1S,3S-ACPD or 2R,4R-APDC) did not produce spontaneous and evoked (mechanical and thermal) nociceptive behaviors in naïve rats [54,68], while a different group II agonist (L-CCG-I, i.th.) had inhibitory effects on the mechanical thresholds (blunt pin) in normal sheep [62] and another group II agonist (DCG-IV) induced pronociceptive responses (paw pressure) in control sham rats [114], suggesting perhaps species differences or non-selective drug actions. Intrathecal application of 2R,4R-APDC inhibited mechanical allodynia (von Frey) in inflammatory (capsaicin, i.pl.) [68] and neuropathic (CCI) pain models [67], but had no effects on capsaicin-induced thermal hyperalgesia [68] or on the CFA-induced mechanical hypersensitivity (von Frey) [115]. Furthermore, DCG-IV (i.th.) ameliorated the mechanical allodynia (von Frey) and hyperalgesia (paw pressure) in neuropathic pain (SNL) rats; these effects were blocked by a group II antagonist (EGLU) [114]. Surprisingly, i.th. administration of a group II mGluR antagonist (LY341495) improved the mechanical allodynia (von Frey) without having effects on thermal hyperalgesia in an inflammatory (CFA) pain model; these beneficial effects were reversed by the co-application of a group II agonist (2R,4R-APDC) and were potentiated by the co-administration of a glial inhibitor (fluorocitric acid) [115]. A few studies addressed the subtype-specific roles of mGluR2 and mGluR3 in the spinal cord. For instance, intrathecal administration of an HDAC inhibitor (suberoylanilide hydroxamic acid, SAHA) reduced the pronociceptive effects of estrogen (E2) on visceral sensitivity (enhanced visceromotor responses to colorectal distension), which was blocked by a group II antagonist (LY341495); SAHA also increased the levels of mGluR2 mRNA and protein in the spinal dorsal horn of ovariectomized rats, pointing to a role of mGluR2 in the epigenetic control of estrogen-related facilitatory effects [116]. On the other hand, i.th. application of NAAG inhibitors (ZJ-11or ZJ-17) reduced the nociceptive behaviors in both phases of the formalin test and mechanical allodynia (von Frey) in the PSNL model, suggesting the involvement of mGluR3 [105]. The mixed roles of spinal group II mGluRs in the behavioral studies (see Table 2) suggest that at the spinal level these receptors may not be a suitable target or perhaps they are involved in more complex pain mechanisms.

Brain. Region-specific differences of the roles of mGluR2/3 in pain modulation have been reported for the brain are conflicting. For instance, stereotaxic administration of a group II mGluR antagonist (EGLU) into the reticular thalamic nucleus, but not other thalamic nuclei, decreased pain behaviors (ankle bend test score) in the CFA-induced monoarthritis pain model [117], while stereotaxic application of a group II mGluR agonist (LY379268) into the amygdala (CeA) decreased the duration of vocalizations in the K/C monoarthritis pain model [97]. Conversely, in the same pain model, the administration of a group II mGluR antagonist (LY341495) into the amygdala (CeA) blocked the inhibitory effects of a systemically applied group II mGluR agonist (LY379268) [97], suggesting that amygdala group II mGluRs may play a crucial role in the beneficial antinociceptive effects of systemic group II mGluR activation perhaps as part of a top-down modulatory pain system. At the level of the brainstem, injection of a group II agonist (L-CCG-I) into dlPAG had pronociceptive effects in the hot plate test under normal conditions [73] but inhibited the nociceptive behaviors in the late phase of the formalin test [74]; these effects were blocked by pretreatment with EGLU in the PAG [73,74]. Microinjection of NAAG or NAAG peptidase inhibitors (JZ-43 or 2-PMPA) into the lateral ventricle (intracerebroventricular, i.c.v.), the locus coeruleus, PAG or RVM decreased pain responses in both phases of the formalin test, implicating mGluR3; and these inhibitory effects were blocked by a group II mGluR antagonist (LY341465) [106,118,119]. Overall, it appears that activation of group II mGluRs in brain regions that engage descending pain modulatory systems (see Table 2) has beneficial effects in pain conditions.

The behavioral studies suggest that the spinal cord may not be the site of action of the beneficial effects of group II mGluRs, whereas the activation of these receptors in supraspinal pain modulatory systems may be a better therapeutical approach for pain relief. More information is needed about subtype specificity to better understand the roles and therapeutic usefulness of mGluR2 and mGluR3 throughout the nervous system. Selectivity of compounds used especially in the earlier studies also needs to be considered.

### 4.2. Preclinical Electrophysiological Studies

Periphery. The application of a group II agonist (2R,4R-APDC) prevented the inflammatory soup- or forskolin-induced thermal sensitization and the capsaicin-induced enhanced excitation of single nociceptive fibers in an ex-vivo rat skin-nerve preparation [107,111]. Additionally, 2R,4R-APDC blocked the membrane hyperexcitability of mouse and human (donors with no history of chronic pain) cultured DRG neurons exposed to PGE_2_ [131] and prevented the PGE_2_ -induced potentiation of capsaicin-evoked Ca^2+^ signals in cultured mouse, but not human, DRG neurons via inhibition of adenylyl-cyclase [109,130]. Group II mGluR antagonists (LY341495 or APICA) enhanced the capsaicin-evoked activity in nociceptive fibers and Ca^2+^ mobilization in dissociated DRG neurons, without having effects alone, supporting the idea that group II mGluRs are endogenously activated in nociceptive sensitization; these responses were abolished by the application of 2R,4R-APDC [112]. In the presence of high concentrations of extracellular glutamate, LY341495 resulted in exacerbated activity and heat responses of nociceptors in the skin-nerve preparation, suggesting that activation of group II mGluRs by exogenous glutamate reduced nociceptor activity [112]. The data generally support an antinociceptive role of peripheral group II mGluRs but subtype-specific functions remain to be determined.

Spinal cord. In spinal cord slices, a group II mGluR agonist (DCG-IV) inhibited Aδ afferent fiber-evoked EPSPs and IPSCs in dorsal horn neurons when applied into the bath under normal conditions [132]; these effects were mediated by a presynaptic action at the excitatory synapses, because group II mGluR activation caused changes in paired-pulse depression and was blocked by a group II mGluR antagonist (EGLU). In vivo single-unit recordings of spinal dorsal horn neurons in anesthetized rats found inhibitory effects of spinal (i.th.) administration of 1S,3S-ACPD on the responses to electrical C-fiber stimulation in an inflammatory pain model (i.pl. carrageenan), but had mixed facilitatory and inhibitory effects in different neurons under normal conditions [82]. Application of group II agonists (LY379268 and L-CCG-I) into the spinal cord abolished the capsaicin-induced sensitization of dorsal horn neurons recorded extracellularly in anesthetized monkeys (Macaca fascicularis), but did not change the responses of non-sensitized neurons to peripheral mechanical stimulation [133]. Interestingly, the inhibitory effects of a systemically (i.p.) applied group II agonist (LY379268) on the evoked responses of spinal dorsal horn neurons in the K/C monoarthritis pain model were blocked by stereotaxic administration of a group II antagonist (LY341495) into the amygdala (CeA) [97]. Accordingly, intra-amygdala (CeA) administration of a group II mGluR agonist (LY379268) reduced the enhanced spinal neuronal activity in arthritic rats (K/C model) [97], suggesting that spinal nociceptive processing can be modulated by group II mGluRs in brain regions such as the amygdala. The data suggest fairly consistent inhibitory effects of group II mGluR activation on spinal neurons in pain conditions, but supraspinal sites of action may be involved and subtype-specific roles remain to be determined.

Brain. In vivo single-unit recordings of amygdala (CeA) neurons in anesthetized rats showed that stereotaxic administration of a group II agonist (LY354740) into the CeA reduced the evoked activity (responses to mechanical stimulation of knee joint and ankle) in normal rats but had more pronounced effects in the kaolin-carrageenan-induced arthritis model [134]. Administration of a group II mGluR antagonist (EGLU) had no effects in normal rats but enhanced the neuronal responses to mechanical stimuli and reversed the inhibitory effects of LY354740 in the arthritis pain model, which is consistent with endogenous group II mGluR activation in pain [134]. Similarly, brain slice physiology recordings of neurons in the CeLC showed that LY354740 decreased excitatory synaptic transmission (EPSCs) at the CeLC-PB synapse under normal condition and more potently in the K/C-induced arthritis pain state through a presynaptic mechanism; these effects were abolished by a group II antagonist (EGLU) [135]; these results were reproduced in a different study showing that a group II agonist (SLx-3095-1), reduced enhanced EPSCs at the PB-CeLC synapse in brain slices obtained from formalin-injected mice [136]. In the IL division of the mPFC, a selective group II agonist (LY379268) inhibited the overall pyramidal cell output by reducing synaptically evoked neuronal spiking in brain slices from normal and arthritis rats (K/C/model) [137]. While LY379268 decreased both EPSCs and IPSCs in either condition, the net effect was inhibition of the mPFC neuronal output because the effects on EPSCs preceded those on IPSCs [137]. Analysis of sEPSCs/mEPSCs and sIPSCs/mIPSCs showed that group II mGluR modulation was due to an action on pre-synaptic glutamatergic, but not GABAergic, terminals. A group II mGluR antagonist (LY341495) abolished the inhibitory effects of the agonist and generated facilitatory responses on synaptically evoked spiking by itself [137]. 

A few studies addressed subtype-specific roles in the brain. The contribution of mGluR2 was studied in the ACC in inflammatory (i.pl. CFA) and neuropathic (CCI) conditions using a transgenic line of *Cre* mice expressing fluorescent protein (td-tomato) in mGluR2 expressing (GRM2) pyramidal neurons. Electrophysiology in mouse brain slices found that application of a group II agonist (2R,4R-APDC) increased rheobase and suppressed the enhanced excitability of sensitized GRM2-td-tomato ACC neurons in both pain models, but had no effects under normal conditions, suggesting that mGluR2 activation can inhibit pain-related hyperexcitability of ACC neurons [138]. The specific role of mGluR3 was studied in the amygdala by testing NAAG and ZJ-43 in brain slice physiology. Under normal conditions, EPSCs at the PB-CeLC synapse were inhibited by NAAG and ZJ-43, mimicking the effects of a group II agonist (SLx-3095-1) mentioned in the previous section. In the formalin pain model, inhibition by ZJ-43 was less effective compared to SXl-3095-1, suggesting reduced NAAG release or increased relative contribution of mGluR2 at the PB-CeLC synapse in the pain condition [136]. The data point to inhibitory functions of group II mGluRs in different brain regions, and behavioral consequence likely depend on their region-specific actions, e.g., amygdala and ACC versus mPFC. The involvement of specific subtypes remains to be determined. 

Compared to their behavioral effects (see Table 2), inhibition of neuronal activity by group II mGluR activation appears to be a more consistent finding. On the other hand, the same neuronal effect in different regions can translate into different, if not opposing, outcomes on pain behavior modulation. Additionally, there is a general lack of subtype-specific mechanisms and actions with some electrophysiological evidence pointing to mGluR2 as a regulator in ACC and amygdala in pain models. 

## 5. Group III mGluRs in Preclinical Pain Models

Group III mGluRs (mGluR4, mGluR6, mGluR7, mGluR8) couple to G_i/o_ proteins to inhibit adenylyl cyclase (thus reducing the formation of cAMP) and protein kinase A activation [139,140]. With the exception of mGluR6, group III mGluRs are mainly expressed presynaptically [141], where they regulate neurotransmitter release [142]. The development of new and more effective compounds for the study of these receptors have greatly helped revealing their physiological role as well as disclosing their therapeutic potential [143]. mGluR4 are predominantly expressed presynaptically [141] and can be found along the pain neuraxis in the DRG [144], spinal cord [145], thalamus [146] and amygdala [12]. mGluR4 mRNA was also found to be moderate in superficial layers of the entorhinal cortex, and weak in the deep layers of the subicular cortex and superficial layers of the parasubicular and presubicular cortices while there was no significant expression of mRNA in the piriform cortex, cingulate cortex, retrosplenial cortex and perirhinal cortex [147]. While mGluR6 is predominantly present in the retina [148], mGluR7 is the most widely expressed subtype in the CNS where they are found at the presynaptic site [146,149]. mGluR8 can be found in the thalamus, cortex and amygdala, where they are localized presynaptically [150] whereas their postsynaptic expression has been reported in the medulla [151] and in the enteric nervous system [152]. 

Generally, activation of group III mGluRs has antinociceptive and beneficial inhibitory effects in pain conditions [5,6]. Group III mGluR agents have been tested systemically (see Table 3). An mGluR4 agonist (LSP4-2022, i.p.) increased mechanical (von Frey) thresholds in the intraplantar carrageenan model of inflammatory pain, without affecting thermal or chemical responses [145]. An mGluR7 PAM (AMN082, i.p.) had no effect in the hot plate and tail flick tests in rats under normal conditions [153] but blocked thermal hyperalgesia and mechanical allodynia in the carrageenan inflammatory model and had beneficial effects on the thermal, but not mechanical, responses in the hindpaw incision model [154]. AMN082 (i.p.) also ameliorated the mechanical withdrawal thresholds and potentiated the beneficial effects of morphine in a neuropathic pain model (CCI) [45]. Somewhat surprisingly, recent studies found that selective NAMs for mGluR7 (MMPIP or ADX71743, s.c.) had antinociceptive properties and mitigated pain-related cognitive deficits in a neuropathic (SNI) mouse model of pain [155] and attenuated visceral hypersensitivity in the colorectal distension test and anxiety-like behaviors in a stress sensitive rat line (Wistar Kyoto rats) [156]. The conflicting effects of pharmacological manipulations of mGluR7 observed in these studies may depend on the route of administration and pharmacological tools; it remains to be determined if and how changes of mGluR7 expression found in neuropathic pain could explain differential pro- and anti-nociceptive actions [6]. Finally, a selective mGluR8 agonist (DCPG, i.p.) had antinociceptive effects by decreasing nociceptive behaviors in early and late phases of the formalin test and improving mechanical and thermal withdrawal latencies in the carrageenan and neuropathic (CCI) pain models [157]. While mGluR4 or mGluR8 activation resulted in antinociceptive responses in different models of pain, mGluR7 pharmacological manipulations yielded contradicting results suggesting more complicated mechanisms in nociceptive function or regions-specific actions or perhaps the involvement of non-neuronal elements (see Ref. [6]).

### 5.1. Preclinical Behavioral Studies

Periphery. Intraplantar injection of a group III mGluR agonist (L-AP4) inhibited nociceptive behaviors of rats in an inflammatory (i.pl. capsaicin) pain model [112]. Somewhat curiously, L-AP4 (i.pl.) blocked the facilitatory responses induced by the application of a group II mGluR antagonist (LY341495, i.pl.) in the capsaicin-injected rats [112]. L-AP4 injected into the knee joint of arthritic rats (intraarticular carrageenan) inhibited secondary mechanical hyperalgesia [113]. Recently, a group III mGluR agonist (L-SOP, i.pl.) was shown to inhibit nociceptive behaviors induced by formalin; this inhibitory effect was abolished by pretreatment with a group III mGluR antagonist (M-SOP) [158]. Therefore, group III mGluR activation in peripheral tissues has beneficial inhibitory effects on pain-related behaviors but information about subtype-specific effects is lacking (see Table 3).

Spinal cord. Early studies have shown that group III mGluR activation with a selective agonist (L-AP4, i.th.) was able to suppress nociceptive responses in the formalin test [63], mechanical and cold hypersensitivity in neuropathic pain models (CCI or SNL) [67,160], and mechanical more than thermal hypersensitivity in the intraplantar carrageenan model [68]. Intrathecal injection of a different group III agonist (ACPT-I) had antihyperalgesic effects in various animal models of inflammatory (CFA-induced monoarthritis, formalin and carrageenan) and neuropathic (CCI and vincristine-induced) pain without affecting nociceptive thresholds in normal animals [159]; these effects were mimicked by the application (i.th.) of a selective mGluR4 PAM (PHCCC) or mGluR4 agonist (LSP4-2022) in the CFA [161], carrageenan and CCI models [145,159]. The beneficial effects of LSP4-2022 (i.th.) were significantly reduced in mGluR4 knockout mice in the intraplantar carrageenan model and by spinal mGluR4 knockdown with i.th. injection of rat mGluR4 antisense oligonucleotides in inflammatory (i.pl. carrageenan) and neuropathic (CCI) pain models compared to mismatched oligonucleotide controls [145], suggesting that spinal mGluR4 activation is antinociceptive and mediates the antinociceptive effects of group III mGluR agonists. Additionally, the reduction of mechanical hypersensitivity observed after intrathecal injection of LSP4-2022 in the CFA inflammatory model was blocked by a photoswitchable mGluR4 NAM (OptoGluNAM4.1, i.th.), which had no effect alone [161]. Spinal mGluR7 also have antinociceptive effects. Intrathecal administration of an mGluR7 agonist (AMN082) inhibited thermal hyperalgesia, but not mechanical allodynia, in the intradermal carrageenan and paw incision pain models [154]. AMN082 (i.th.) also blocked the development of mechanical and thermal hypersensitivity in neuropathic rats (paclitaxel model) and decreased glial reactivity in the spinal cord and the release of pro-inflammatory cytokines in the paclitaxel-induced pain model [162]. The behavioral beneficial effects of AMN082 were reversed by the intrathecal co-administration of a mGluR7 NAM (MPIP) [162]; these data suggest consistent antinociceptive functions of spinal group III mGluRs (mGluR4 and mGluR7) (see Table 3).

Brain. Microinjection of a group III mGluR agonist (L-SOP) into the dlPAG produced a dose-dependent decrease of nociceptive latencies in the hot plate test, and pretreatment with a selective group III mGluR antagonist (MSOP) abolished these pronociceptive effects and increased the latencies when administered alone in normal mice [73]. To date, a few studies have explored the subtype-specific group III mGluR functions in brain regions in pain conditions. An active photoswitchable mGluR4 PAM (optogluram) injected into the amygdala inhibited mechanical allodynia (von Frey) in an inflammatory pain model (intraplantar CFA) similar to the intra-amygdalar administration of an mGluR4 agonist (LSP4-2022). The antiallodynic properties were abolished by the inactivation of optogluram with violet light illumination into the amygdala [12]. The activation of mGluR7 may be pro- or anti-nociceptive, depending on its primary site of action [124]. For instance, a pronociceptive role of mGluR7 is supported by the finding that intra-amygdala (CeA) application of an mGluR7 agonist (AMN082) decreased mechanical thresholds, increased vocalizations to mechanical compression of the knee joint, and induced anxiety-like behaviors (elevated plus maze) in normal rats [164]. Similarly, AMN082 injected into the PAG or the dorsal striatum was also found to be pronociceptive as it decreased the thermal latencies (tail flick test) and mechanical thresholds under normal conditions [165,166]. In contrast, AMN082 had antinociceptive effects when injected into the nucleus tractus solitarius and nucleus accumbens [167,168] in normal animals. Mixed effects have been reported in pain condition. Intra-dorsal striatum injection of AMN082 had antinociceptive properties in neuropathic (SNI) rats [166] whereas intra-amygdala (CeA) administration of AMN082 had no effect on mechanical hypersensitivity, emotional responses and anxiety-like behavior in the K/C arthritis rat model [164]. Administration of a selective mGluR8 agonist (DCPG) or mGluR8 PAM (AZ2216052) into the PAG, dorsal striatum or amygdala (CeA) increased thermal (tail flick and plantar test) and mechanical withdrawal thresholds in inflammatory (carrageenan) and neuropathic (SNI) conditions [157,163,169], although intra-PAG administration of DCPG was recently reported to have no effects on thermal hyperalgesia [171] but decreased cold allodynia [172] in a neuropathic pain model (spinal cord contusion). The beneficial effects of the DCPG in the CeA or PAG were blocked by the co-administration of a group III mGluR antagonist (MSOP) [157,163], which did not change the nociceptive responses when administered alone in inflammatory pain (carrageenan) or normal conditions [163]. Strangely, application of a presumed mGluR6 agonist (Homo-AMPA) into the PAG had antinociceptive effects, decreasing mechanical allodynia (von Frey) and increasing thermal latency (tail flick) in normal and diabetic neuropathic (streptozotocin model) conditions, although mGluR6 mRNAs and proteins were not detected in the PAG, suggesting that Homo-AMPA may not be a selective agonist for mGluR6 but may involve potential activation of mGluR8, which still remains to be tested [170]; these findings also confirm that GluR6 expression is predominantly outside of the central nervous system [148]. Overall, the data suggest that mGluR4 and mGluR8 have more consistent inhibitory effects on pain behaviors than mGluR7, which can have region-specific pro- and antinociceptive functions (see Table 3).

The behavioral data suggest that systemic activation of group III mGluRs, including mGluR4 and mGluR8, has antinociceptive effects in pain conditions, whereas both activators and inhibitors of mGluR7 function were antinociceptive. While mGluR4 activation in spinal and supraspinal regions has consistently been shown to inhibit pain behaviors, the beneficial effects of mGluR8 may reside with actions in the brain (information about spinal mGluR8 is lacking). The mixed pattern of mGluR7 functions observed with systemic activation may be due to pro- and anti-nociceptive effects, or lack of effects, found with mGluR7 activation in different brain regions and under normal conditions versus pain models, although antinociceptive effects of spinal mGluR7 have been reported quite consistently; it should be noted that currently no information is available about subtype-specific functions of peripheral group III mGluRs in pain behavioral modulation. Potential region-specific actions of individual subtypes emphasize the need for studies to address this knowledge gap.

### 5.2. Preclinical Electrophysiological Studies

Periphery. Little information is available about electrophysiological effects of peripheral group III mGluRs in pain; however, in a recent study, electrophysiological single-unit recordings showed that local administration of a group III mGluR agonist (L-AP4) into the inflamed (CFA) knee joint attenuated neuronal excitability of mechanosensitive C- and Aδ-afferent fibers measured as discharge frequency evoked by the mechanical stimulation of the knee with von Frey filaments. Importantly, the beneficial effects were not observed in the control (sham) group, providing evidence for a pain-specific role of peripheral group III mGluRs [173]. A group III mGluR antagonist (UBP1112) enhanced the capsaicin-mediated facilitation of single nerve fibers and Ca^2+^ mobilization in dissociated DRG neurons without having effects by itself; these responses were abolished by bath application of L-AP4 [112]. The data suggest that peripheral group III mGluRs can inhibit afferent activity and may be activated endogenously in pain conditions.

Spinal cord. In vivo extracellular single-unit recordings from spinal dorsal neurons in anesthetized monkeys (Macaca fascicularis) showed that spinal application of L-AP4 inhibited the responses to cutaneous mechanical stimuli in a concentration-dependent manner under normal conditions and blocked the enhanced responses induced by capsaicin injection into the foot [133]. In spinal cord slice preparations, L-AP4 suppressed A-fiber-evoked excitatory postsynaptic potentials (EPSPs) [132] and primary afferent-evoked EPSCs and IPSCs [174] recorded in dorsal horn neurons under normal conditions. Greater effects were found in spinal slices obtained from neuropathic (SNL) rats [174]. The pharmacological effects of L-AP4 were mediated by presynaptic group III mGluRs as revealed by the analysis of paired-pulse depression (PPD) [132] and mEPSCs/sEPSCs and mIPSCs/sIPSCs [174]. An mGluR4 agonist (LSP4-2022) reduced the amplitude of evoked EPSCs in dorsal horn (lamina II) neurons in spinal cord slices from normal mice, and more strongly, in slices from mice with hindpaw inflammation (CFA) through a mechanism that involved the inhibition of voltage-gated N-type (Cav2.2) calcium channels in the presynaptic terminal [145]. The findings are consistent with the behavioral data showing consistent antinociceptive functions of spinal group III mGluRs, including mGluR4.

Brain. In vivo electrophysiological recordings of individual neurons in the amygdala (CeA) in anesthetized rats found that stereotaxic administration of a group III agonist (L-AP4) into the CeA reduced the evoked activity (responses to mechanical stimulation of knee joint and ankle) in normal rats but had more pronounced effects in the kaolin-carrageenan arthritis model [134]. A group III mGluR antagonist (UBP1112) had no effects under normal conditions but increased the evoked responses in the arthritis pain condition, suggesting increased endogenous activation [134]. In whole-patch clamp experiments in rat brain slices, L-AP4 had inhibitory effects on excitatory synaptic transmission at the PB-CeLC synapse, decreasing the amplitude of EPSCs more potently in brain slices from arthritic rats than control rats while increasing paired-pulse ratio without affecting neuronal excitability or slope conductance [175]. The inhibitory effects of L-AP4 were blocked by the application of a group III mGluR antagonist (UBP1112) [175]. The effects of L-AP4 were mimicked by a selective mGluR8 agonist (DCPG), which inhibited excitatory, but not inhibitory, transmission at the BLA-CeLC synapse more potently in brain slices from arthritic rats (K/C model) than in control rats through a presynaptic mechanism based on sEPSC and mEPSC analysis [176]. In contrast, a selective mGluR7 agonist (AMN082) increased excitatory transmission in slices from normal rats but not arthritic rats through the inhibition of synaptic inhibition, likely involving BLA-activated GABAergic neurons in the intercalated cell masses (ITC) [176]. The selective activation of mGluR4 with LSP4-2022 decreased the EPSCs of the dorso-medial ITC cells and principal neurons of the lateral amygdala (LA) evoked by electrical or optogenetic (ChR2) stimulation of the thalamic inputs, and had a facilitatory effect on the paired-pulse ratio, suggesting a presynaptic action of mGluR4 at thalamus-LA and thalamus-ITC synapses [12]. A selective mGluR8 antagonist (MDCPG) injected into the LEC induced long-term potentiation at the LEC-DG pathway by enhancing the amplitude and slope of fEPSPs in SNI mice chronically treated with vehicle or PEA and prevented the further potentiation of the fEPSPs after TBS in neuropathic (SNI model), but had no effects under normal conditions [93]. 

In vivo electrophysiological single-unit recordings of RVM neurons showed that mGluR7 and mGluR8 activation in the PAG differentially modulates the activity of RVM ON (“pronociceptive”) and OFF (“antinociceptive”) cells, a key network of the descending pain modulatory system [165]. Specifically, activation of mGluR8 in the PAG with DCPG reduced the ongoing activity of RVM ON cells while increasing the activity of OFF cells in anesthetized normal rats. On the other hand, mGluR7 activation in the PAG with AMN082 increased the spontaneous neuronal firing of RVM ON cells and decreased the activity of OFF cells, providing evidence for differential functions of mGluR7 and mGluR8 in pain control [165]. The effects of DCPG or AMN082 were reversed by the pretreatment with a group III mGluR antagonist (MSOP) that had no significant effect by itself [165]. The opposite effects of mGluR7 and mGluR8 activation observed in the in vivo electrophysiological studies of RVM cells correlated with the analysis of neurotransmitter levels in the dialysates collected from the PAG in freely moving animals. AMN082 decreased glutamate release but did not change GABA levels in the PAG [165] whereas DCPG increased glutamate and decreased GABA release in the PAG [177]. In neuropathic (SNI) rats, blockade of mGluR7 in the PAG by a selective mGluR7 NAM (MMPIP) reduced ON cell activity and enhanced the OFF cell activity [155]. MMPIP applied systemically (i.p.) restored the imbalance between excitation and inhibition of PL cortical neuronal activity evoked by BLA electrical stimulation in SNI mice [155]. More recently, the application of AMN082 in the dorsal striatum was shown to have pronociceptive effects by enhancing the spontaneous and tail flick-evoked activity of the RVM ON cells while inhibiting those activities in OFF cells in normal animals and opposite effects were found in the neuropathic (SNI) condition, providing additional evidence for dual effects of mGluR7 on nociceptive processing in the brain [166]. In contrast, mGluR8 activation by DCPG in the dorsal striatum increased ongoing activity of RVM OFF cells and inhibited that of ON cells in rats with SNI. DCPG also reduced tail flick-induced ON cell burst and OFF cell pause and increased the onset of both ON cell burst and OFF cell pause in the same animals [169]. The electrophysiology data are consistent with the results from behavioral studies pointing to more consistent pain inhibitory effects of mGluR4 and mGluR8 than mGluR7 activation in limbic and pain control brain regions.

In summary, evidence from behavioral (see Table 3) and electrophysiological studies suggests that group III mGluR activation can inhibit pain processing and pain behaviors and these beneficial effects involve primarily mGluR4 and mGluR8 rather than mGluR7, although mGluR7 activation in the spinal cord has demonstrated antinociceptive effects. Little is known about the role of peripheral group III mGluR subtypes in pain modulation.

## 6. Role of mGluRs in Neuroimmune Signaling

A rapidly growing domain of pain research focuses on the contribution of non-neuronal factors to pain development and maintenance. Many recent reviews have highlighted the influence of intricate crosstalk between neurons, immune cells, and glial cells in pain-related peripheral and central sensitization [178,179,180,181,182,183], though little investigation has focused on pain-related neuroimmune mechanisms within the brain. Further, the relationship between mGluRs and neuroimmune signaling as a mechanism of neuroplasticity and chronic pain has not been explored systematically. Though most predominantly expressed on neurons, mGluRs are also widely found on neuroimmune cells such as astrocytes and microglia [184]. Whereas astrocytes under physiological conditions regulate homeostatic control of CNS environment through the removal of glutamate from the extracellular space and the pruning of neural synapses [185,186], reactive astrocytes may take on a dysfunctional role in pathological states by precipitating neuroinflammation and excitotoxicity [187,188]. Similarly, microglia function as resident macrophages of the CNS by pruning synapses and phagocytosing cells under normal conditions [189,190], though they become activated in response to pathological stimuli and release factors that also contribute to a neuroinflammatory state [191,192]. As astrocytes and microglia likely influence pain-related neuroplasticity at both spinal and supraspinal levels [193,194], the mechanistic role of glial mGluRs in this process is a critical avenue of future exploration.

Changes in glial mGluR signaling have been implicated in various neurological and neurodegenerative diseases outside of the pain field. Perhaps most notably are the contributions of mGluR3 and mGluR5, which are expressed in glial cells throughout the brain [195,196,197,198,199] and are highly upregulated in reactive astrocytes [200,201,202]. Despite coupling to G proteins of opposing functions, these receptors may act synergistically in the developing mouse brain [203]. There, activated mGluR3 may potentiate mGluR5-induced calcium transients to increase overall astrocytic intracellular calcium release, which promotes structural and functional maturation in sensory networks [203]. In pathological states, however, the role of these receptors may vary. In cultured astrocytes and microglia from an experimental model of amyotrophic lateral sclerosis (ALS), treatment with the pro-inflammatory cytokines tumor necrosis factor α (TNFα) and interleukin 1β (IL-1β) induced an increase in mGluR3 but a decrease in mGluR5 gene expression [204]; however, in the hSOD-G93A rat ALS model, a robust upregulation of mGluR5 was seen in cultured mutated astrocytes compared to wild-type, though stimulation of the receptor failed to activate astrocytic glutamate uptake [205]. Another study showed that knockdown of mGluR5 in SOD1-G93A mouse ALS model decreased astrocyte and microglia activation, normalized glutamate release in the spinal cord, and improved motor skills during disease progression, though interestingly the latter effect occurred in male but not female mice [206]. Together these data suggest that mGluR5 may contribute to glutamate excitotoxicity in ALS pathogenesis and blocking this receptor may represent a therapeutic strategy for this disease. Similarly, in the transgenic APP/PS1 mouse model of Alzheimer’s disease, mGluR5 overexpression has been demonstrated on reactive astrocytes surrounding β-amyloid (Aβ) plaques [207], and in a cell culture system, the binding of (Aβ) oligomers to the astrocytic cell surface triggered the clustering of mGluR5 receptors and a resultant increase in adenosine triphosphate (ATP) release following activation of astroglial mGluR5 by its agonist (DHPG) [207]. Therefore, mGluR5 may also play a significant role in ATP-induced glutamate release that contributes to neurodegeneration in Alzheimer’s disease [208,209]. The contribution of glial mGluRs has also been shown to extend to addiction pathogenesis. Injection of the toll-like receptor 3 (TLR3) agonist polyinosinic:polycytidylic acid (poly I:C), a potent immunostimulant and viral mimetic, increased mRNA expression of mGluR2 (and glutamate transporter 1, GLT1), but not mGluR3 and mGluR5, in the rat insular cortex (IC) and nucleus accumbens (NAc), two brain regions shown to modulate ethanol drinking [210]. Systemic delivery of a group II mGluR agonist (LY379268, i.p.) reduced ethanol self-administration in poly I:C treated animals but not in controls, supporting the theory that neuroimmune activation leads to increases in glutamatergic function that affect ethanol sensitivity, which can be reduced by group II mGluR activation [210]. Together, these studies illustrate known interactions between mGluRs and neuroimmune signaling in various neurological pathologies, which may also be relevant for pain states.

Despite this support for a neuroimmune component of mGluR signaling in other pathologies, little is known about glial mGluRs in the field of pain research. In the CCI model of neuropathic pain, elevated expression levels of mGluRs (mGlu5 and mGlu3, but not mGlu7, mRNA; mGluR5, but not mGluR2/3 and mGluR7, proteins) were seen in the ipsilateral spinal cord, and administration (i.p.) of glial inhibitors (minocycline and pentoxifylline) prevented these injury-induced expression changes [211]; these findings suggest that the majority of upregulated mGluR5 receptors after injury are located on glial cells, as the pharmacological inhibition of glia eliminated most of the changes in mGluR5 mRNA and protein levels [211]. Though mGluR5 is normally absent in healthy adult astrocytes [212,213,214], it may transiently reemerge within astrocytes of the somatosensory (S1) cortex under pathological conditions such as chronic pain [215]. One group reported a temporary (less than 10 day) upregulation of mGluR5 in astrocytes of the contralateral S1 cortex following PSNL that was absent in sham operated animals; this limited reemergence of astrocytic mGluR5 increased calcium signaling, upregulated synaptogenic molecules to induce mechanical allodynia, and persistently enhanced S1 neuronal activity, all of which was abolished following astrocyte-specific mGluR5 deletion [215]; this suggests that astrocytic mGluR5 may function as a critical controller of synaptic plasticity in S1 cortical networks under pain conditions. With regard to mGluR7, another group found that an mGluR7 PAM (AMN082) inhibited glial reactivity and decreased pro-inflammatory cytokine release in paclitaxel-induced acute neuropathic pain; this inhibition of the spinal glial reaction alleviated mechanical and thermal hypersensitivity in neuropathic rats, providing further support for a neuroimmune role of mGluR signaling in pain pathogenesis [162]. Despite the rapid growth of novel findings with regard to neuroimmune-related pain processing, mechanisms of mGluR modulation have yet to be clearly characterized. As demonstrated by exploration of glial mGluRs in other disease states, these receptors may also represent novel therapeutic targets for pain management. 

## 7. Sex Differences and mGluRs

It is important to note that an increasing amount of evidence suggests there are many sexually dimorphic mechanisms that contribute to pain development and maintenance (recently reviewed in [216]). As female subjects have historically been overwhelmingly understudied in both the preclinical and clinical setting, there is a lack of full understanding of sex-specific processes at all levels of the neuraxis. Studies in other neurological disciplines have begun to investigate potential sex differences related to mGluRs; it is well established that estrogen receptors can couple with mGluRs to initiate G protein signaling cascades that influence neuronal activity and behavior [217,218,219]; this signaling has been shown to affect reward circuitry and motivational behavior in females [220], drug addiction in females [221], endocannabinoid system regulation in females [222], opioid analgesic mechanisms in females [223], and antinociception across the female reproductive cycle [224]; however, it is unclear whether hormonal contributions fully encompass sex-specific mechanisms of mGluR signaling. In a rat model of prenatal chronic mild stress (PCMS), mGluR expression patterns showed significant region-dependent sex differences following PCMS induction, with a male-specific upregulation of mGluR2/3 and mGluR5 in the PFC [225]. Following PCMS coupled with acute stress from the forced swim test (FST), male PMCS-FST rats showed lower levels of mGluR5 in the hippocampus, lower mGluR5 but higher mGluR2/3 in the PFC, and higher mGluR5 levels in the amygdala than control males; however, female PMCS-FST rats only differed from control with regard to a lowered level of mGluR2/3 in the amygdala [225]. Male rats that were subjected to prenatal restraint stress (PRS) showed increased anxiety-like behavior in the elevated plus maze (EPM) with a reduction of group I mGluR activity in the ventral hippocampus, whereas female PRS rats showed reduced anxiety-like behavior in the EPM and an increase in group I mGluR activity in the same region [226]; these data suggest that stress can induce sex-specific changes in glutamatergic (including mGluR) signaling that may influence behavioral outcomes in males and females. 

Sex-dependent influences on other mGluRs have also been reported in the anxiety field. In mGluR8-deficient adult mice, males showed increased but females showed decreased anxiety-like behaviors in the EPM and in the acoustic startle response; this deficiency also impaired spatial memory retention in the Morris water maze to a greater extent in female than male mice [227]. Within the amygdala, ovariectomized rats with both high and low estradiol levels had significantly higher protein levels of mGluR1 than male rats. Infusion of a group I mGluR agonist (DHPG) into the BLA, a brain region implicated in anxiety responses (and pain processing), produced anxiolytic-like effects in ovariectomized female rats but anxiogenic-like effects in male rats [228]. As pain and anxiety are often comorbid, the two states may share neurobiological mechanisms in brain regions such as the amygdala that contribute to pain modulation and emotional network plasticity [84,229,230,231]. Despite this well-established link, very little has been investigated with regard to sex-specific mechanisms of mGluR signaling in pain-related processing. In the formalin pain model, administration of a selective serotonin reuptake inhibitor (SSRI) produced analgesic responses only in female mice and increased the protein expression of mGluR2 in the spinal dorsal horn, possibly via the reduction of histone deacetylase 2 (HDAC2) in DRGs [232]. Together, the current literature provides ample support for the need to examine mGluR signaling mechanisms in both sexes to gain a more complete understanding of pain modulation throughout the neuraxis.

## 8. Conclusions

The preclinical behavioral and electrophysiological studies reported in this review generally support an important role of different mGluRs in pain processing and pain modulation in several models, providing a strong rationale for exploring the therapeutical potential of compounds acting on mGluRs (Figure 1). Currently, there is insufficient information to arrive at definitive conclusions about which mGluR subtype(s) may represent the most preferable approach to mitigate pain. With the development of more selective molecules (PAMs and NAMs) to target specific mGluR subtypes, significant progress has been made in our understanding of mGluRs and mGluR subtypes, but more work is needed to address the contributions of specific mGluR subtypes at different levels of the pain neuraxis (periphery, spinal cord, brain) to pain processing and pain modulation (Figure 1). Still, the evidence reviewed here suggests an important contribution of mGluR1 to pain mechanisms whereas the role of mGluR5 is more complex and region specific; mGluR1 blockade would be a desirable strategy. The situation is less clear for group II mGluRs. Behavioral studies point to beneficial effects of activation of these receptors at supraspinal sites whereas electrophysiological data show more consistent inhibitory effects in the periphery, spinal cord and brain regions, but these could translate into differential behavioral consequences. There is no clear picture with regard to mGluR2 and mGluR3 subtypes. Beneficial effects of group III mGluR activation is supported by behavioral and electrophysiological studies and there is evidence to suggests that these involve primarily mGluR4 and mGluR8 but mGluR7 activation in the spinal cord has demonstrated antinociceptive behavioral effects. In general, allosteric modulators seem to have beneficial effects in pain conditions, providing compelling evidence for their potential employment in clinical trials. Important knowledge gaps that need to be addressed urgently include the role of different mGluRs and mGluR subtypes in pain-related sex differences and their role in the neuroimmune mechanisms of pain. 

## Figures and Tables

**Table 1:** Behavioral effects of group I mGluR drugs in preclinical pain models.

Receptor	Drug Type	Compound	Route of Administration	Species	Pain Model	Effect	References
Systemic							
mGluR1	NAM	EMQMCM	i.p.	Rat	Normal	No effect	[37]
					Formalin	Inhibition	
		FTIDC	i.p.	Mouse	Formalin	Inhibition	[38]
		A-841720, A-794282, A-794278, A-850002	i.p.	Rat	Skin incision, MIA, CFA, CCI, SNL	Inhibition	[39,41]
		LY456236	i.p.	Mouse	Formalin	Inhibition	[40]
				Rat	SNL		
mGluR5	NAM	MPEP, MTEP	i.p., oral	Rat	Normal	No effect	[37,42]
			i.v.			Inhibition	[46]
		Fenobam, MPEP, MTEP	i.p.	Mouse	Formalin, CFA	Inhibition	[26,27,38]
			Oral, i.p.	Rat	Formalin, CFA, carrageenan, CCI, SNL, visceral, skin incision		[28,37,40,42,43,44,45]
Periphery							
mGluR1/5	Agonist	DHPG	i.pl.	Rat	Normal	Facilitation	[47,48]
				Mouse			[49]
mGluR1	NAM	CPG, CPCCOEt, LY367385	i.pl.	Mouse	Normal	No effect	[49]
				Rat			[48]
			Masseter muscle		Normal, exogenous glutamate, DHPG or CHPG-induced hyperalgesia		[50]
			i.pl.				[47]
				Mouse	Formalin	Inhibition	[49]
					Capsaicin		
				Rat			[48]
					IL-1β-injection		[51]
mGluR5	Agonist	CHPG	i.pl.	Rat	Normal	Facilitation	[47]
	NAM	MPEP, SIB1893	i.pl.	Mouse	Normal	No effect	[49]
				Rat			[48]
			Masseter muscle		Normal, exogenous glutamate, DHPG or CHPG-induced hyperalgesia	Inhibition	[50]
			i.pl.				[47]
					CFA, carrageenan		
					Skin incision		[52]
				Mouse	Formalin		[49]
					Capsaicin		
				Rat			[48]
					IL-1β-injection		[51]
	Photoswitchable NAM	JF-NP-26	Light-mediated activation on the paw	Mouse	Formalin	Inhibition	[53]
Spinal							
mGluR1/5	Agonist	DHPG, 1S,3R-ACPD	i.th.	Rat	Normal	Facilitation	[54,55,56,57,58]
				Mouse			[59,60,61]
				Sheep			[62]
				Mouse	CFA		[60]
				Rat		Inhibition	[58]
					Formalin	Facilitation	[63]
	Antagonist	LY393053, S-4C3HPG, AIDA	i.th.	Rat	SNI, CFA, CCI	Inhibition	[64,65,66,67,68]
mGluR1	NAM	CPCCOEt	i.th	Mouse	Formalin	Inhibition	[59]
				Rat	CCI		[56,67]
mGluR5	NAM	Fenobam, MPEP	i.th.	Mouse	Formalin	Inhibition	[59]
				Rat	SNI, CFA, CCI, paclitaxel, streptozotocin		[56,64,65,67,69,70]
Brain							
mGluR1/5	Agonist	DHPG	CeA	Rat	Normal	Facilitation	[71,72]
			dlPAG				
				Mouse			[73]
					Formalin	Inhibition	[74]
	Antagonist	AIDA	dlPAG	Mouse	Normal	Inhibition	[73]
mGluR1	NAM	CPCCOEt	CeA	Rat	K/C-monoarthritis	Inhibition	[75]
			BLA		Carrageenan		[76]
mGluR5	NAM	MPEP, MTEP	CeA	Rat	K/C-monoarthritis	Inhibition	[75]
			Dorsal striatum			No effect	
			BLA		Carrageenan		[76]
			mPFC		SNL	Inhibition	[77]
	Photoswitchable NAM	Alloswitch-1	Amygdala	Mouse	CFA	Inhibition	[78]
		JF-NP-26	Light-mediated activation in the VPM of the thalamus		CCI, formalin		[53]
	Lentivirus	EF1α-mGluR5-IRES-Zsgreen1	mPFC	Rat	Normal	Inhibition	[77]
	PAM	VU0360172	mPFC	Rat	K/C-monoarthritis	Inhibition	[79]
			ACC			No effect	

**Table 2:** Behavioral effects of group II mGluR drugs in preclinical pain models.

Receptor	Drug Type	Compound	Route of Administration	Species	Pain Model	Effect	References
Systemic							
mGluR2/3	Endogenous activation	NAC	i.p.	Mouse	Normal	Inhibition	[94]
					Formalin, CCI, CFA		[95]
	Agonist	LY379268, LY354740, LY389795		Rat	Normal	No effect	[96,97]
					Formalin, carrageenan, SNL, K/C-monoarthritis	Inhibition	[96,97,98]
				Mouse	CCI		[45]
		LY2969822	Oral	Rat	Formalin, capsaicin, CFA, SNL, skin incision, visceral pain		[99]
				Mouse	Acid acetic writhing		
mGluR2 knock-out	Endogenous activation	NAC	i.p.	Mouse	Formalin	Inhibition	[95]
mGluR3 knock-out	Agonist	LY379268				No effect	[100]
mGluR2	Receptor expression Potentiation	LAC	s.c., oral	Rat	Normal	Inhibition	[101]
					CCI		
					Carrageenan (synergism with PEA)		[102]
				Mouse	CFA, streptozotocin		[103]
		SAHA	s.c.		Formalin		[104]
mGluR3	Receptor activator (NAAG inhibitor)	ZJ-11, ZJ-43 or 2-PMPA	i.p., i.v.	Rat	Formalin, SNL	Inhibition	[105,106]
Periphery							
mGluR2/3	Agonist	LY314582, 2R,4R-APDC, L-CCG-I, SLx-3095-1, DCG4	i.pl.	Rat	Normal	No effect	[47,48,107,108]
				Mouse			[109,110]
				Rat	Capsaicin	Inhibition	[111,112]
					Carrageenan		[108]
					Formalin		
					Infl. soup		[107]
					IL-1β- injection		[51]
			Knee joint		Carrageenan-induced monoarthritis		[113]
			i.pl.	Mouse	Carrageenan, PGE2-injection		[109,110]
	Antagonist	APICA	i.pl.	Rat	Capsaicin	Facilitation	[112]
		LY341495		Mouse	Carrageenan, PGE2-injection		[110]
mGluR3	Receptor activator (NAAG inhibitor)	ZJ-43, 2-PMPA	s.c.	Rat	Carrageenan, formalin	Inhibition	[108]
Spinal							
mGluR2/3	Agonist	L-CCG-I	i.th.	Sheep	Normal	Inhibition	[62]
		DCG-IV		Rat		Facilitation	[114]
					SNL	Inhibition	
		1S,3S-ACPD, 2R,4R-APDC			Normal	No effect	[54,68]
					CFA		[115]
					Capsaicin		[68]
						Inhibition	
					CCI		[67]
	Antagonist	LY341495	i.th.	Rat	CFA	Inhibition	[115]
						No effect	
mGluR2	mGluR2 expression (HDAC inhibitor)	SAHA	i.th.	Rat	Estrogen injection	Inhibition	[116]
mGluR3	Receptor activator (NAAG inhibitor)	ZJ-11, ZJ-17	i.th	Rat	Formalin, PSNL	Inhibition	[105]
Brain							
mGluR2/3	Agonist	L-CCG-I	dlPAG	Mouse	Normal	Facilitatory	[73]
					Formalin	Inhibition	[74]
		LY379268	CeA	Rat	K/C- monoarthritis	Inhibition	[97]
	Antagonist	EGLU	Reticular nucleus of the thalamus	Rat	CFA	Inhibition	[117]
			Paratenial nucleus of the thalamus, medial corticohypothalamic tract region			No effect	
mGluR3	Receptor activator (NAAG inhibitor)	JZ-43, 2-PMPA	i.c.v.	Rat	Formalin	Inhibition	[118]
			Locus coeruleus				[106]
			PAG, RVM				[119]

**Table 3:** Behavioral effects of group III mGluR drugs in preclinical pain models.

Receptor	Drug Type	Compound	Route of Administration	Species	Pain Model	Effect	References
Systemic							
mGluR4	Agonist	LSP4-2022	i.p.	Rat	Carrageenan	Inhibition	[145]
mGluR7	PAM	AMN082	i.p.	Rat	Normal	No effect	[45,154]
					Carrageenan, skin incision, CCI	Inhibition	
	NAM	MMPIP,	s.c.	Mouse	SNI	Inhibition	[155]
		ADX71743		Rat	Visceral pain		[156]
mGluR8	Agonist	DCPG	i.p.	Mouse	Carrageenan, formalin, CCI	Inhibition	[157]
Periphery							
mGluR4/6/7/8	Agonist	L-AP4, L-SOP	i.pl.	Rat	Formalin	Inhibition	[158]
					Capsaicin		[112]
			Knee joint		Carrageenan		[113]
Spinal							
mGluR4/6/7/8	Agonist	L-AP4, ACPT-I	i.th.	Rat	Carrageenan	Inhibition	[68,159]
					Formalin		[63,159]
					CFA		[159]
					Vincristine-injection		
					SNL		[160]
					CCI		[67,159]
mGluR4	Agonist	LSP4-2022	i.th.	Mouse	Carrageenan	Inhibition	[145]
				Rat	Normal	No effect	
	PAM	PHCCC			Carrageenan, CCI	Inhibition	[159]
	Photoswitchable NAM	OptoGluNAM4.1	i.th.	Mouse	CFA	No effect	[161]
mGluR4 knock-out	Agonist	LSP4-2022	i.th.	Mouse	Carrageenan	No effect	[145]
mGluR4 knock-down	Antisense oligonucleotides (i.th.)	LSP4-2022	i.p.	Rat	Carrageenan	Slight inhibition	[145]
mGluR7	PAM	AMN082	i.th.	Rat	Carrageenan, skin incision	Inhibition	[154]
					Paclitaxel		[162]
Brain							
mGluR4/6/7/8	Agonist	L-SOP	dlPAG	Mouse	Normal	Facilitation	[73]
	Antagonist	MSOP	CeA	Rat	Normal	No effect	[163]
					Carrageenan		
mGluR4	PAM	LSP4-2022	Amygdala	Mouse	CFA i.pl.	Inhibition	[12]
	Photoswitchable PAM	Optogluram					
mGluR7	PAM	AMN082	CeA	Rat	K/C- monoarthritis	No effect	[164]
					Normal	Facilitation	
			dlPAG				[165]
			Dorsal striatum				[166]
					SNI	Inhibition	
			Nucleus of the solitary tract		Normal	Inhibition	[167]
			Nucleus accumbens				[168]
mGluR8	Agonist	DCPG	dlPAG	Mouse	Carrageenan	Inhibition	[157]
			CeA	Rat	Carrageenan		[163]
			Dorsal striatum		SNI		[169]
					Normal	No effect	
	PAM	AZ2216052	Dorsal striatum	Rat	Normal	No effect	[169]
					SNI	Inhibition	
mGluR6	Agonist	Homo-AMPA	dlPAG	Mouse	Normal	Inhibition	[170]
					Streptozotocin		
